# Hard plant tissues do not contribute meaningfully to dental microwear: evolutionary implications

**DOI:** 10.1038/s41598-019-57403-w

**Published:** 2020-01-17

**Authors:** Adam van Casteren, David S. Strait, Michael V. Swain, Shaji Michael, Lidia A. Thai, Swapna M. Philip, Sreeja Saji, Khaled Al-Fadhalah, Abdulwahab S. Almusallam, Ali Shekeban, W. Scott McGraw, Erin E. Kane, Barth W. Wright, Peter W. Lucas

**Affiliations:** 1 Department of Anthropology, Washington University in St. Louis, St. Louis, MO 63130 USA; 2 Department of Bioengineering, Don State Technical University, Rostov-on-Don, Russia; 3 Department of Bioclinical Sciences, Faculty of Dentistry, Kuwait University, P.O. Box 24923, Safat, 13110 Kuwait; 4 Department of Mechanical Engineering, College of Engineering and Petroleum, Kuwait University, P.O. Box 5969, Safat, 13060 Kuwait; 5 Department of Chemical Engineering, College of Engineering and Petroleum, Kuwait University, P.O. Box 5969, Safat, 13060 Kuwait; 6 Department of Anthropology, 4064 Smith Laboratory, The Ohio State University, 174 West 18th Ave., Columbus, OH 43210-1106 USA; 7 Department of Anthropology, Boston University, 232 Bay State Rd, Boston, MA02215-1403 USA; 8 College of Osteopathic Medicine, Kansas City University of Medicine and Biosciences, 1750 Independence Ave., Kansas City, MO 64106 USA; 9 Smithsonian Tropical Research Institute, Apartado Postal, 0843-03092 Panamá, República de Panamá; 10 Palaeo-Research Institute, University of Johannesburg, Auckland Park, Gauteng, South Africa

**Keywords:** Biomechanics, Biological anthropology

## Abstract

Reconstructing diet is critical to understanding hominin adaptations. Isotopic and functional morphological analyses of early hominins are compatible with consumption of hard foods, such as mechanically-protected seeds, but dental microwear analyses are not. The protective shells surrounding seeds are thought to induce complex enamel surface textures characterized by heavy pitting, but these are absent on the teeth of most early hominins. Here we report nanowear experiments showing that the hardest woody shells – the hardest tissues made by dicotyledonous plants – cause very minor damage to enamel but are themselves heavily abraded (worn) in the process. Thus, hard plant tissues do not regularly create pits on enamel surfaces despite high forces clearly being associated with their oral processing. We conclude that hard plant tissues barely influence microwear textures and the exploitation of seeds from graminoid plants such as grasses and sedges could have formed a critical element in the dietary ecology of hominins.

## Introduction

Early hominin craniodental morphologies, evolving before cooking or sophisticated extra-oral food processing, represent adaptations to diet, but profound disagreement persists about the specific foods that drove evolutionary change. Isotopic evidence demonstrates that, starting in the mid-Pliocene (circa 3.5 million years ago) and continuing into the Pleistocene, the composition of hominin diets broadened. In most hominin species, it shifted over this period from consumption almost exclusively of C_3_ vegetation (circa 85% of diet) to encompassing a moderate-to-large proportion of C_4_ plant material (35–77% of diet)^1,2^. Although isotopic evidence indicates the photosynthetic pathway of carbon fixation, these data do not directly indicate the exact dietary source of such a signal. Enriched carbon implies early hominins ate either C_4_ grasses, sedges or the animals that consumed these same graminoid plants^1,3^. However, predictions of what plant part may have contributed to such a signal vary: some authors suggest leaves^1,3^, while others focus on energy-rich plant storage organs such as corms or bulbs^4^. Here we advocate the case for seeds. These various plant parts differ in their mechanical properties and thus promote contrasting selection pressures on tooth morphology.

**Figure 1:**
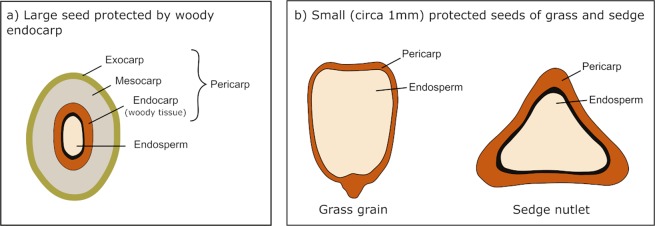
A schematic drawing of seeds mechanically protected by lignified woody tissue. (**a**) Large seeds of some dicotyledonous plants are protected by a woody seed shell. (**b**) Even small seeds of monocotyledons have lignified pericarps protecting the seed within.

Mechanical analyses of australopith teeth and jaws indicate that they were capable of generating high bite forces^5,6^ with their very thick tooth enamel both strengthening their teeth and prolonging their functional life^7–9^. In particular, the low blunt cusps of australopith molars would be more resistant to fracture against hard foods, as exemplified by the woody casings of what are thus ‘mechanically protected’ plant embryos (Fig. 1)^10^. Whether this casing is derived from the seed integuments or the fruit endocarp, we call this a ‘mechanically-protected seed’ here. This feeding association contrasts with primates that typically have long sharp crests on their teeth as adaptations to eat tough compliant foods like leaves^7,8,11,12^. However, the morphological signal in australopiths seems to be at odds with the microwear signal conveyed in the surface texture of wear facets in hominin teeth.

Conventional interpretations of dental microwear in primates suggest that a diet consisting of a large proportion of hard objects would produce a surface texture with a high complexity. Complexity is essentially a measure of the surface roughness, with wear facets demonstrating high complexity often associated with deep and elaborate scars^13^. Although there is variation in the microwear signals of Plio-Pleistocene hominins, in general, the wear facets of most early hominin teeth exhibit low to moderate complexity^1,14^. One notable exception from this trend is that of *Paranthropus robustus* which in some cases exhibits high surface texture complexity^1^. However, most australopiths tend to exhibit light surface striations that, in several species, are not strongly directed in parallel^1^. This lack of surface texture complexity is more in keeping with what one would expect from a primate that eats a considerable amount of tough compliant material, such as leaves, although extant primate folivores tend to exhibit surface textures with parallel oriented striations^15^. The apparent mismatch between morphology and microwear has fuelled a continual and at times heated debate about early hominin diets. Nowhere is this disparity more salient than for *Paranthropus boisei* whose highly derived, robust morphology has earned it the epithet “nutcracker man”, stemming from a predicted diet laden with hard objects^5^. Yet microwear studies have indicated this same species did not routinely eat hard objects and that its dentition was used for the processing of softer, tougher foods^3^. Resolution of these radically different interpretations requires an evaluation of the mechanics of microwear formation.

Currently, there are little to no experimental data on sliding contacts between particles of woody plant material, such as pieces of seed shell, and enamel. Such particles represent the hardest plant tissues and, based on mechanical models of wear^16^, such tissues should not impart much damage on teeth. However, if particles of lignified plant tissue are unable to produce the deep or elaborate scars on enamel it seems unlikely that feeding on dietary items, such as mechanically protected seeds, would produce the complex surfaces textures predicted by traditional interpretations of dental microwear. It is plausible then that the presence or absence of complex surface textures measured in microwear analysis of tooth facets may not directly reflect the consumption of hard foods, but instead echo levels and types of dietary abrasives^17^.

Here we present data on nanowear experiments investigating the interaction between heavily lignified plant tissue and enamel. We demonstrate that although the densest woody tissue can mark enamel surfaces it cannot produce deep elaborate features on the tooth surfaces. Further, by combining these data with compressive tests of some of the smallest hard C_4_ seeds, we show that high forces can be generated during the oral milling of large quantities of them. Such orofacial forces may provide a selective force driving the evolution of robust craniodental morphology in early hominins.

## Results

### Sliding experiments

**Figure 2:**
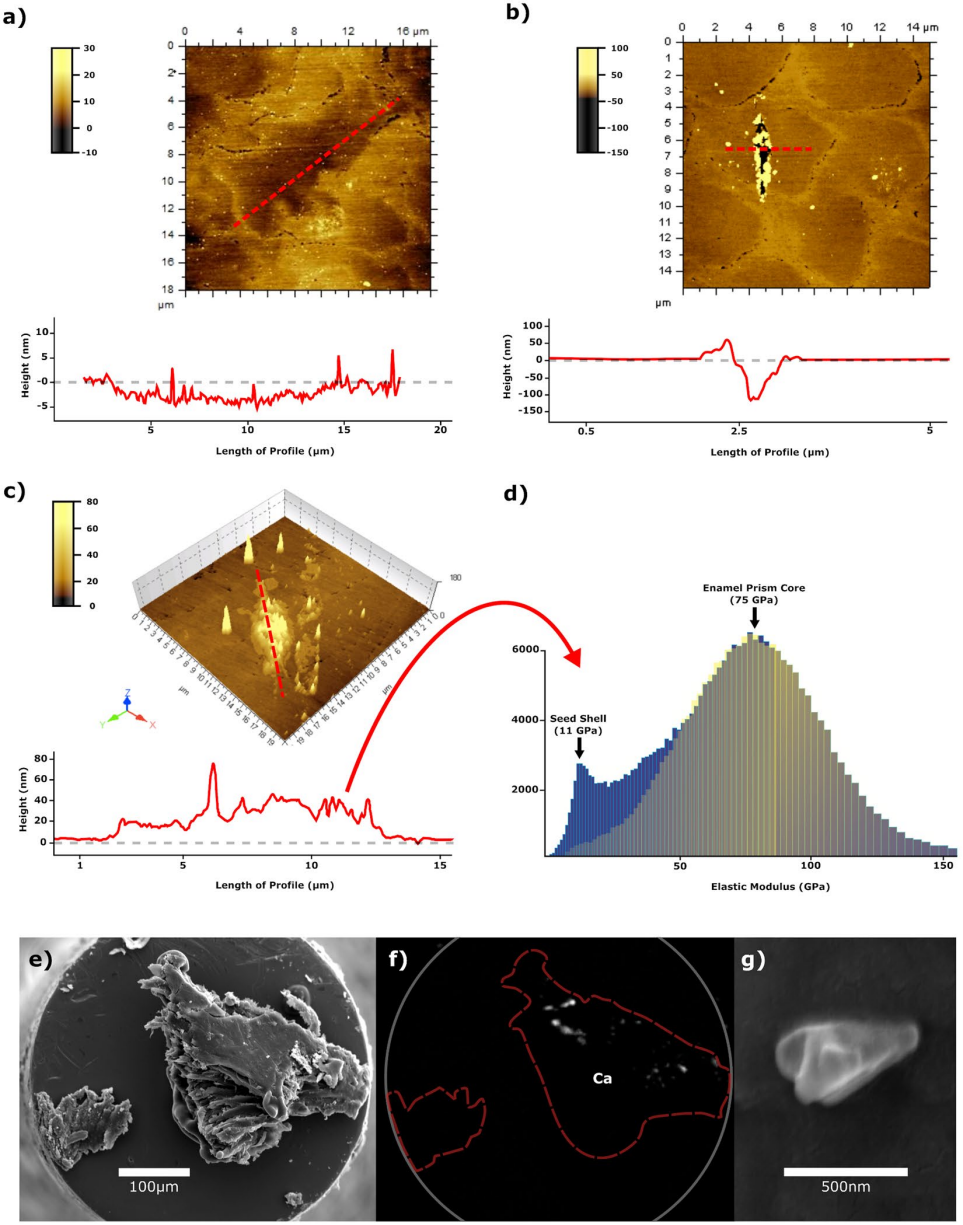
AFM topography traces of a tooth surface (shading indicates depth in nm) around detectable damage following sliding contacts against seed shell fragments. (**a**) *Elaeis guineensis*, accompanying graph is a 3D longitudinal profile of the enamel mark, which is just 5 nm deep with a length of ~15 µm. (**b**) *Sacoglottis gabonensis* with accompanying cross-sectional profile. This was the most pronounced mark recorded during the experiments. (**c**) *Mezzettia parviflora* where the accompanying longitudinal profile highlights deposits of material in the damage zone. (**d**) Using the AFM as nanomechanical force microscope, the deposit in **c** is shown to have the elastic modulus of the seed shell of *M. parviflora*. (**e**) SEM micrograph of piece of *Sacoglottis gabonensis* seed shell on end of a flat-head indenter, post-test. (**f**) Energy dispersive spectroscopy (EDS) maps of this shell fragment, post-test; small amounts of calcium are present. (**g**) Example of the extremely small enamel chips found adhering to the woody tissue.

We performed single-slide nanowear experiments^16^ (total slides n = 16) making contacts between fragments of three woody seed shells (Fig. 2a–c) present in primate diets (*Elaeis guineensi*s, Arecaceae, *Sacoglottis gabonensis*, Humiriaceae; *Mezzettia parviflora*, Annonaceae) against enamel at forces between 0.4–1.2 mN, varied at 0.2 mN intervals. The nanohardness measured for these woody seed shells is typical of other protective endocarps and the pericarp of grasses and sedges, but very high in relation to plant tissues generally^12^. However, they are an order of magnitude lower in hardness than that of either dental enamel or phytoliths (Table 1).

**Table 1:** Comparison of mechanical properties pertinent to wear of woody plant tissue versus tooth enamel, phytoliths and quartz grit.

Material ID(*ref*)	Species of Seed (family)	Hardness (GPa)	Elastic Modulus (GPa)	Toughness (MPa.m^½^)
**Tooth Enamel**^31^				
Orangutan	*Pongo pygmaeus* (Hominidae)	5.0	100	2.5
**Small Seeds**^5^				
C_4_ grasses	*Sporobolus spicatus* (Poaceae)	0.125	3	—
	*S. ioclados*	0.295	4.7	—
	*Pennisetum stramineum*	0.56	7	—
	*Cynodon dactylon*	0.175	3.2	—
	*Eragrostis sp*.	0.25	3.7	—
C_4_ sedge	*Cyperus bulbosus* (Cyperaceae)	0.16	3.6	—
C_3_ sedge	*Carex monostachya*	0.585	6.2	—
**Large Seeds**				
Orangutan food^19,32^	*Mezzettia parviflora* (Annonaceae); shell zone II	0.49	9.4	3.5
Sooty mangabey food	*Sacoglottis gabonensis* (Humiriaceae)	0.27	8.2	1.4
Chimpanzee food	*Elaeis guineensis* (Arecaceae)	0.4	9.6	—
Hunter-gatherer food	*Sclerocarya birrea* (Anacardiaceae)	0.265	9.1	—
Hunter-gatherer food	*Ricinodendron rautanenii* (Euphorbiaceae)	0.26	11.1	2.1
**Mineral**^16,33^				
Phytoliths	*Ampelodesmos mauritanicus* (Poaceae)	2.8	20	0.22
	*Dactylis glomerata* (Poaceae)	3.0	22	0.28
Kuwait landscape	Quartz	13.5	95	0.7

Hardness and elastic modulus were obtained via nanoindentation; toughness values are from macroscale tests.

There was no evidence of large pits or scratches/fractures of the enamel (Fig. 2a–c), such as produced by some extraneous grit/dust particles^16^. There was also no evidence on the enamel of the ‘prow’, produced by contact with phytoliths^16^. Shallow grooves in the enamel, maximally 150 nm deep and less than a micron wide, were sometimes observed (31% of contact events). One groove was observed for both *E. guineensis* and *S. gabonensis* and three grooves were observed for *M. parviflora*. These markings were similar in length to the slide displacement, in the same direction, and thus undoubtedly caused by them (Fig. 2a–c). They were much less pronounced than marks produced by phytoliths in similar sliding experiments^16^ and would barely register in dental microwear texture analyses as conventionally performed^15^. We expect that even dozens of these marks on a standard scale dental microwear surface would manifest themselves as gentle ripples rather than a highly complex texture.

In experiments performed on *M. parviflora* seed shell, mounds of material were deposited on the enamel (Fig. 2c) that were large enough to investigate. Using an atomic force microscope (AFM) as a bimodal nanomechanical force microscope^18^, the elastic modulus of these deposits was shown to be ≈11 GPa (Fig. 2d), similar to seed shell values^19^ and much lower than that of enamel (median 75 GPa). Thus, rather than woody plant material abrading enamel, the converse occurs with enamel escaping relatively unscathed.

Results from the EDS mapping of the piece of seed shell after the sliding experiment did reveal very small (submicron) enamel chips on its exterior (Fig. 2f,g). Technically, enamel tissue loss of this kind is defined mechanically as abrasion, but the scale of tissue removal was much smaller than that caused by mineral particles of similar size^16^.

### Seed compression and CT scanning

**Figure 3:**
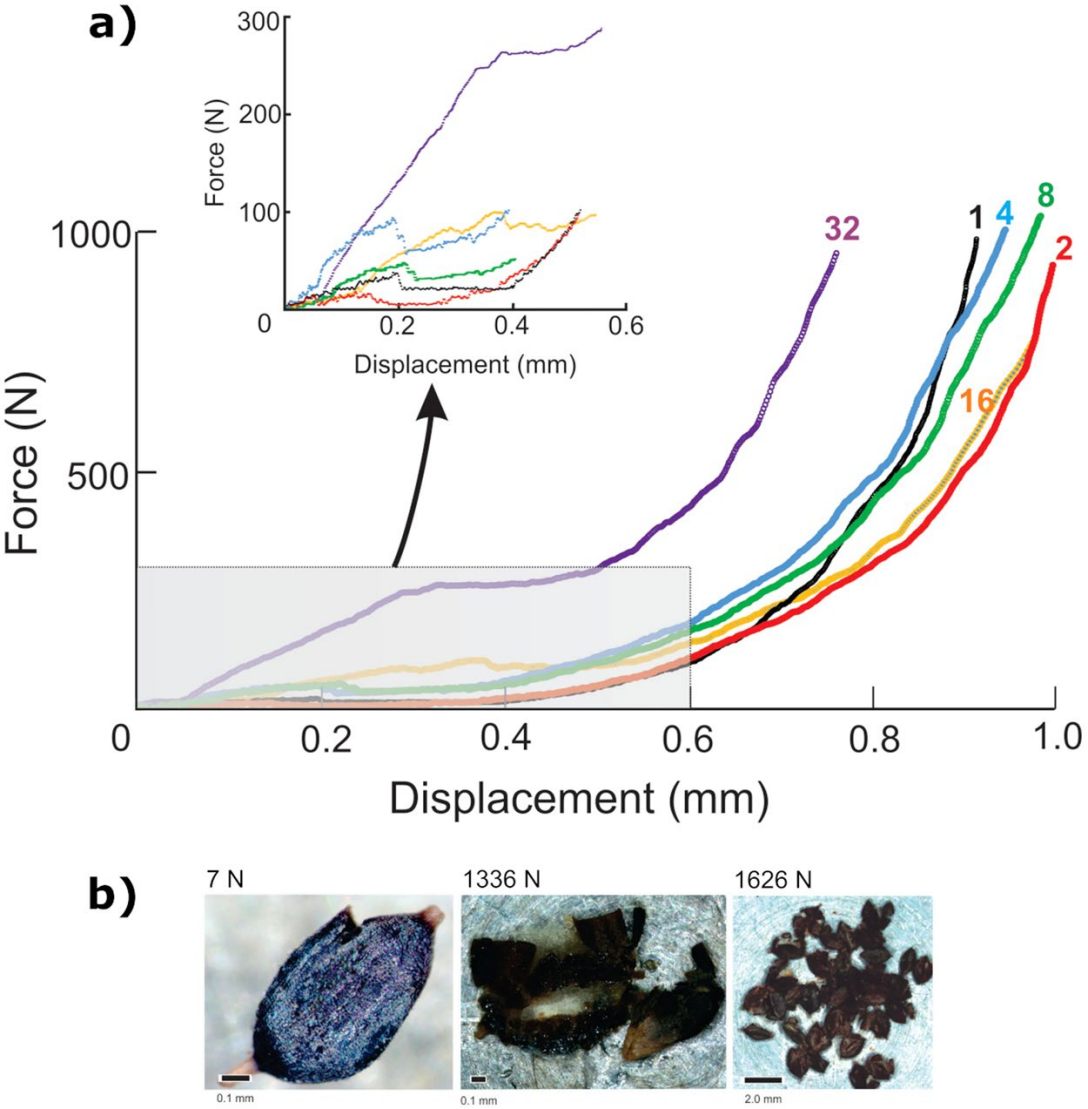
Results from sedge nutlet compression tests. (**a**) Force-displacement plots for compression of differing numbers of nutlets of a sedge, *Carex monostachya*. Initial fracture occurs at a small force, but further compression releases the endosperm at far higher forces, a pattern amplified as more nutlets are compressed. (**b**) Images demonstrating catastrophic nutlet failure needed to access the nutrient-rich tissues are a function of both force magnitude and the number of nutlets. High forces are needed for nutlet densification, yet for any given force some nutlets may not fail catastrophically if many nutlets are consumed at once. This implies that processing many nutlets at once requires high forces or repetitive loading.

We compressed samples of sedge nutlets (where, throughout, ‘nutlet’ refers here to the formal botanical term for the fertilized fruit of sedges), showing that high forces can be reached during mastication of even a small number of them (Fig. 3a). Initial fracture of their pericarp starts at low force (possibly lower with an additional lateral force) yet persists as further loading opens the pericarp completely to facilitate chemical access to the nutritious interior (Fig. 3b). The enclosed endosperm densifies as the load increases and the gradient of the force-displacement curve elevates dramatically. Simple mechanics predict that these forces rise with an increase in the number of nutlets processed. Natural variation in individual nutlets complicates experimental results, but in our experiments the trial with the greatest number of nutlets registered the highest forces.

**Figure 4:**
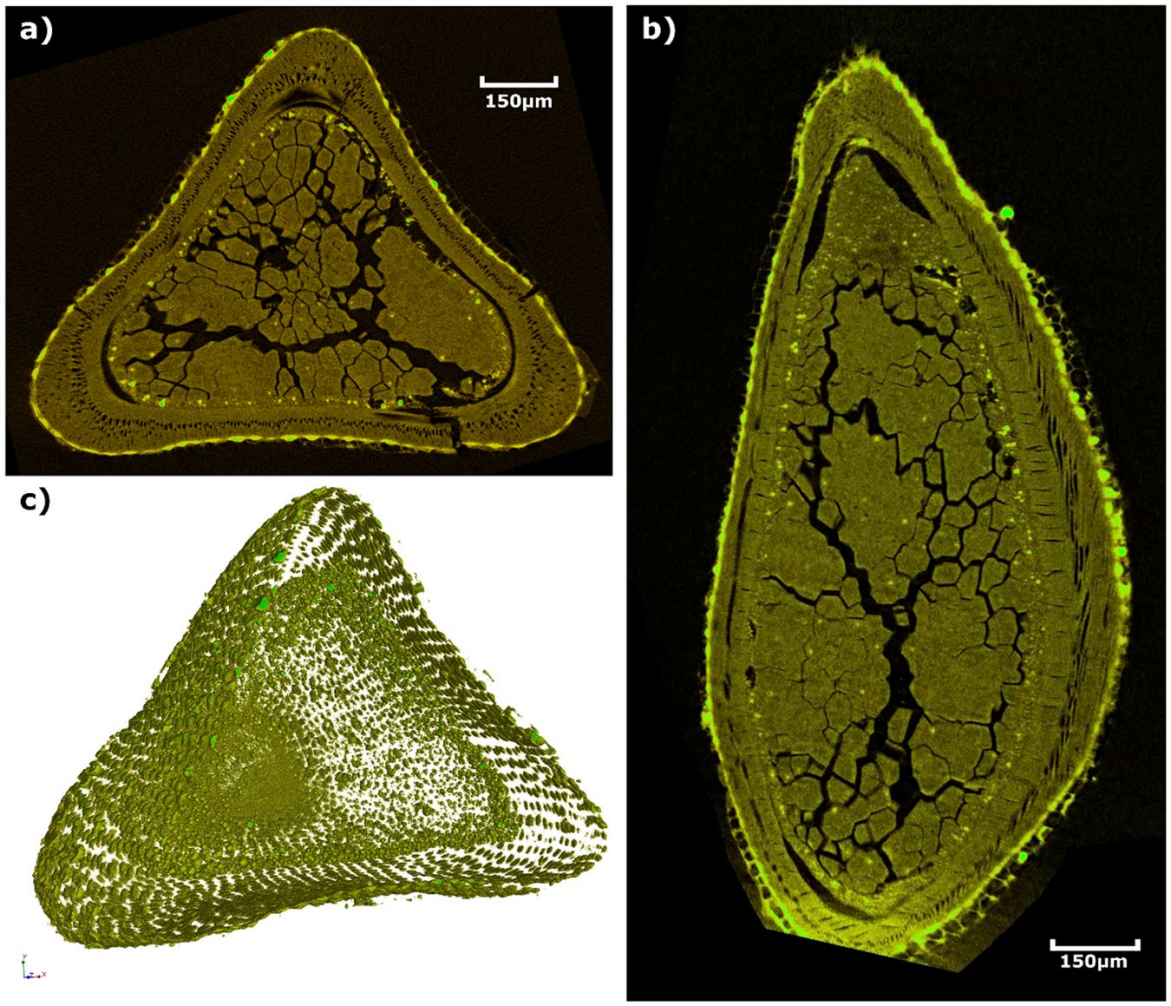
Micro-CT scans of a *Carex monostachya* nutlet. (**a**) A transverse slice and (**b**) a longitudinal slice through the nutlet; phytoliths show up as green flecks. When segmented (**c**), large numbers of phytoliths are seen in both seed coat and outer regions of the endosperm, highlighting their position and distribution within the nutlet.

Micro-CT scans of a *Carex monostachya* nutlet revealed that it is populated by numerous phytoliths (Fig. 4 and Video S1, green flecks). When segregated it was apparent that phytoliths can be found abundantly in the outer pericarp, but some are located too on the inner endosperm.

## Discussion

Our experiments demonstrate that contact between woody tissue and teeth did not directly produce the deep and substantial pitting that leads to complex microwear textures on primate teeth. Such limited damage is consistent with mechanical models of tooth wear that predict when sliding contacts between a “hard” particle and a tooth surface occur there can be two main resulting actions: “rubbing” or “abrasion” (also sometimes termed “cutting”)^16^. During rubbing, no material is instantly lost throughout contact between a surface and a sliding particle. Instead, the material on the surface is merely rearranged producing a shallow groove with noticeable pile up of displaced material at the edges. Contrary to rubbing, abrasive actions, defined here as the removal of dental material in a single tribological event via cutting or chipping from the surface, produce a deep v-shaped scratch mark^16,20^. The distinction as to whether sliding contacts between a particle and surface produce either rubbing or abrasion is dictated by the particle mechanical properties and the critical angle of attack. If a particle has a sufficient hardness, and the attack angle is also above the critical angle (dictated by the toughness of the surface), then material will be removed, leaving behind a scar in the form of an irregular pit or an angular scratch. If however the particle is of a lower hardness, and/or the angle of attack is below the critical value, then material will be plastically rearranged^16^, leaving behind a furrow with a smooth cross-section.

Previous experiments with enamel have shown that hard geologically-derived particles like quartz can easily produce substantial abrasive scratches on teeth. However small “hard” plant-derived particles like phytoliths lack the mechanical hardness to produce abrasive marks and instead only rub enamel. This produces visible damage to the surface but does not remove material instantly from the tooth^16^. Whilst repeated rubbing might cause eventual material loss, this will be at a much-reduced rate compared to harder particles like quartz. Lignified plant tissue being considerably softer than either quartz or phytoliths (Table 1) would by mechanical predictions impart limited damage to enamel. Our experiments confirmed this at the scale of microwear with such contacts generally producing no large identifiable marks on enamel. We do not doubt that occasional contacts between seeds and irregular spicules of enamel could result in the latter being fractured, but over time this process should result in a decrease, rather than increase, in texture complexity. As a generalization, seeds cannot be the source of complex textures conventionally attributed to hard object feeding.

It has been proposed^3^ that food material properties may indirectly influence microwear patterns because of how the jaw movements of a primate are modulated when eating hard versus tough, softer foods. According to this kinematic hypothesis, as hard food items are compressed between teeth moving vertically towards each other, hard particles are driven vertically into the occlusal surface to produce pits. Similarly, as tough, softer foods are trapped between tooth surfaces that are sliding past each other during large transverse jaw displacements, hard particles should be dragged across the tooth surface producing linear scratches aligned in the direction of the jaw movement. Mechanical experiments in which grit is processed by flat tooth surfaces that either slide transversely across or compress vertically towards each other seem to corroborate this hypothesis^21^. However, jaw movements in living primates do not vary so much as to be purely transverse or purely vertical. Moreover, *in vivo* chewing experiments on humans and capuchin monkeys show that the consumption of hard foods is associated with greater transverse jaw movements than when eating tough, softer foods^22,23^. These data thus contradict a key premise of this kinematic hypothesis. Further, this hypothesis relies on the assumption that any “hard” particle can cause substantive abrasive damage to enamel. The results we present here confound such assumptions by demonstrating that some of the hardest plant foods are incapable of producing the characteristics of complex surface textures in enamel.

We favor an alternative model of interpreting microwear^17^. Namely, mastication of thin, film-like tissues (like leaves or grass blades) ought to produce microgrooves aligned in the same direction because phytoliths or grit particles will contact tooth surfaces only as opposing dental contact facets are sliding past one another (which can happen without large jaw excursions). In contrast, mastication of thicker or isodiametric tissues may produce irregular contacts between particle and occlusal surface as the food is rolled between the teeth, or as the food tissues are laterally displaced as the food item is vertically compressed. If mineral grit is present during these contacts, then complex pitting might ensue. If grit is absent but phytoliths are present, then rubbing marks might be produced, but the features would not be aligned. Thus, *contra* conventional wisdom, microwear analysis of surface textures may not provide direct evidence about food material properties, but rather inform on interactions between particle shapes and sizes in the mouth, as well as the relative proportions of hard, angular abrasive particles (such as quartz and silicates) versus phytoliths that can produce initially non-abrasive surface yielding on enamel.

If lignified plant tissues cannot severely damage enamel at the microwear scale, challenging conventional interpretations of dental microwear, then the lack of complexity in enamel surface textures no longer rules out hard object feeding as a significant component of australopith diets. Grass or sedge seed consumption is consistent with the moderate-to-high C_4_ isotopic signal recovered from the teeth of many australopiths since many African grasses and sedges are C_4_ plants. Ecological considerations suggest that such seeds could have served as an important, seasonally available food source capable of contributing substantially to the energetic needs of a large bodied hominin. Previous research into African tropical grasslands suggests that seed production would be seasonal, usually occurring around 3 months after the onset of rain and persisting until the end of a rainy season delivering a productive period that may span 4–5 months^24^. The reproductive effort of grass is linked to rainfall. Grass seed production varies widely between plant species, but in general a crop of 10^3^–10^4^ seeds/m^2^ has been proposed for tropical grasslands^24^. The mass of a grass seed varies greatly and is dependent on species. However, a mean seed weight can be calculated from 10 African grass species as 0.00037 g (dry wt. basis)^24^. Taking this as typical, then it can be estimated that 1 m^2^ of tropical grassland could produce between 0.37–3.7 g of seed. Grass seeds are considered energy-rich with 1 kg of grass seed proposed to deliver an estimated 15 MJ in energy^25^ - more than enough to support a large bodied ape and even a modern human^26,27^. This being so, (assuming a daily energy budget for a pre-*Homo* hominin as circa 6.3 MJ; ref. ^27^), then a patch of tropical grassland potentially as small as 135 m^2^ could provide enough energy to sustain such a hominin daily. There is no living analogue for the diet that we presume australopiths may have been consuming, but the behavior of geladas^28^ and yellow baboons^29^ show some similarities, the latter consuming the seeds of two C_4_ grasses that we have studied. Yet it is clear that the consumption of large amounts of grass and sedge seeds would require both high magnitude and highly repetitive loading to break the protective woody exteriors, while daily foraging could be achieved with quite limited ranging.

Grass and sedge seed consumption is also consistent with cusp and tooth crown morphology. Yet, one might ask whether the consumption of such small seeds would require adaptations to produce high bite forces. Compression tests of sedge nutlets indicate that high forces can be reached during mastication of even a small number of them (Fig. 3a). A large-bodied hominin would be required to orally mill large amounts of sedge and grass seeds to fulfil its daily energetic needs, meaning that the consumption of small mechanically protected seeds in large numbers should reasonably require high-magnitude repetitive forces. This could impart large, continuous stresses to the molar teeth, requiring thickened enamel to resist fractures and maintain functional efficiency for as long as possible. Moreover, phytoliths in the casings of some such seeds (Fig. 4) could explain the presence of light grooves on the dental microwear textures of many early hominins, and the lack of alignment of such grooves could be explained by the transverse displacement of parts of the pericarp away from the seed centroid (in a manner analogous to the lateral displacement of the sides of a solid as it is axially compressed) as the endosperm is densified. Phytoliths in the pericarp would therefore be moving in all directions parallel to the occlusal surface, resulting in relatively isotropic microwear textures.

Our analyses show that the hardest plant tissues produce markings on enamel surfaces that cannot be directly responsible for pitting and surface complexity; at most, these tissues produce light rubbing marks. We further show that small mechanically protected grass and sedge seeds can require high forces to process orally. We conclude that consumption of grass and sedge seeds is compatible with the available data on australopith diets and feeding adaptations and hypothesize that such foods were a selectively important component of early hominin diets. Such selection pressures were effectively side-stepped with sophisticated extra-oral processing and cooking practices in later hominins, but prior to this, small-object feeding, once thought to be a driver of hominin adaptations^30^, seems entirely plausible.

## Materials and Methods

### Nanowear experiments and imaging

The enamel sample was taken from a museum specimen of a Bornean orangutan (*Pongo pygmaeus*) molar tooth. This molar was donated to PWL by the Raffles Museum of Biodiversity Research in Singapore (permission granted by its then director Mrs Yang Chang Man); it is the same as used in previous nanowear studies^16^. The molar was sectioned longitudinally and the enamel surface polished down to a 20 nm r.m.s. surface roughness using colloidal silica between each seed shell experiment. The tooth enamel was not fresh or maintained in a hydrated state for this experiment. Recent research has indicated that dehydrating enamel reduces the tissue’s ability to resist fracture^29^. Therefore, our experiments represent a conservative estimate of the conditions needed to induce mechanical damage in enamel as fresh, hydrated enamel will likely be more durable. The nanohardness of both the enamel and sections of the three seed shells was measured with a Berkovich tip (Hysitron Ubi1, Minneapolis, MN, USA).

Given the irregular shapes of the seed shell fragment, to ensure scratch damage could be located, a ‘landing strip’ was created on the enamel surface by indenting a Berkovich diamond tip into the enamel surface at 8 mN to produce two parallel lines: four indents on one side, roughly 20 µm apart and three indents on the other, with an 80 µm wide landing strip between them. Searches for marks were made within these strip boundaries. This working area was located not on occlusal enamel but between the occlusal surface and the enamel-dentine junction (EDJ). Although the mechanical properties of dental enamel have been shown to vary in some species from EDJ to the occlusal surface, in *Pongo* this difference is limited^31^. Additionally, if the initial occlusal surface were somehow adapted to be more wear resistant, then by conducting tests deep to this surface we again ensure our results are a conservative estimate of the conditions needed to induce mechanical damage in enamel with plant material.

Fragments of woody seed shell were made by pressing a large seed section against a serrated blade. From the debris, for each plant species an appropriately sized particle was chosen and fixed to a custom manufactured flat-headed titanium tip using cyanoacrylate glue (Fig. 2e). Light microscopy was used to verify that the seed shell fragment was properly attached and free from adhesive on the contacting surface. The tip and affixed particle were then placed into the nanoindenter for sliding experiments to be performed. Contact between particle and enamel consisted of a lateral displacement at 10–15 µm at fixed vertical forces increasing by 0.2 mN intervals between 0.4–1.2 mN. These forces were chosen as they correspond to previous experiments on microwear formation^16^, where it was shown that other dietary abrasives (grit, phytoliths and enamel chips) could inflict markings on enamel surfaces. Such minute forces are far below maximum bite forces for any primate, so marks produced in this experimentation could be reproduced in almost any masticatory action.

After each particle of seed shell was slid at the various forces, the landing strip was searched for evidence of damage using an atomic force microscope (5500 AFM, Agilent, Santa Clara, CA, USA) in tapping mode in sequential 80 × 80 µm scans. When damage was identified, higher magnification scans of the area of interest were generated allowing high resolution images and 3D profiles of scratch zones to be analyzed.

When there was clear evidence of debris within the strip, the elastic modulus of this debris was determined by an AFM (MFP-3D, Asylum, Oxford Scientific, UK) configured as a bimodal nanomechanical force microscope^18^. The elastic moduli of both debris and enamel surface, standardized to soda lime glass (*E* = 70 GPa), were recorded. Data scatter reflects, in part, the hierarchical composite nature of these materials.

### EDS mapping

Both pre- and post-test, the elemental composition of each seed shell fragment tip was mapped using energy dispersive spectroscopy (EDS, Oxford Instruments, Abdingdon, UK) attached to a scanning electron microscope (SEM, Jeol 7001F, Tokyo, Japan), a combination that allows high-resolution imaging and elemental mapping. When calcium was present in post-test scans, it was possible to correlate the image with the submicron enamel chips, using their identity.

### Sedge nutlet compression and CT scanning

Small sedge nutlets were compressed between flat plates at speeds of 1–2 mm min^−1^ and the resultant force and displacements recorded by a materials tester (FLS-1 tester, Lucas Scientific, New York, USA) fitted with a 2 kN load cell. CT images of intact seeds were made on a GE Phoenix Nanotom M (Wunstorf, Germany). Scans were displayed at a resolution of 0.93 µm/voxel using a voltage of 100 kV at a current of 260 µA. Total scan time was 250 min.

## Supplementary information

Supplementary Movie S1.

## Data Availability

The data that support the findings of this study are available from the corresponding author upon reasonable request.
